# Mediastinal tumor: not always a lymphoma

**DOI:** 10.1590/S1679-45082017AI3981

**Published:** 2017

**Authors:** Silvia Mansur Reimão, Rogério Colaiacovo, Marco Antonio Ribeiro Camunha, Thiago Trolez Amancio, Vanderlei Segatelli, Gustavo Andrade de Paulo

**Affiliations:** 1 Hospital Israelita Albert Einstein, São Paulo, SP, Brazil.

**Figure 1:**
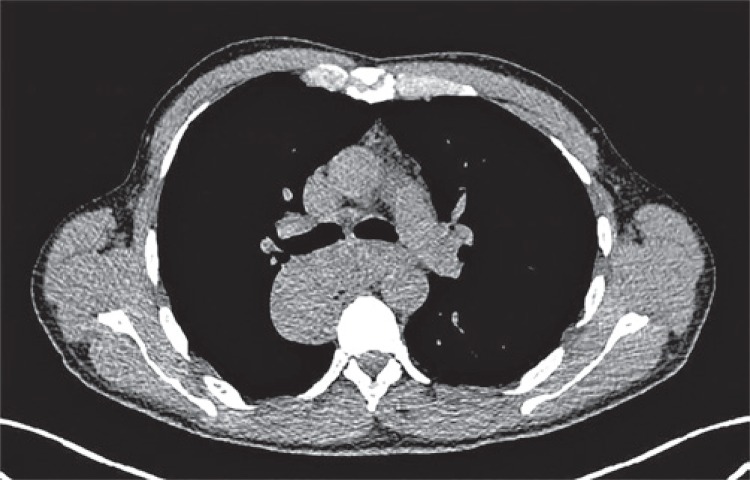
Computerized tomography. Lesion in the posterior mediastinum in contact with the main bronchi, descending aorta, and azygos vein

**Figure 2:**
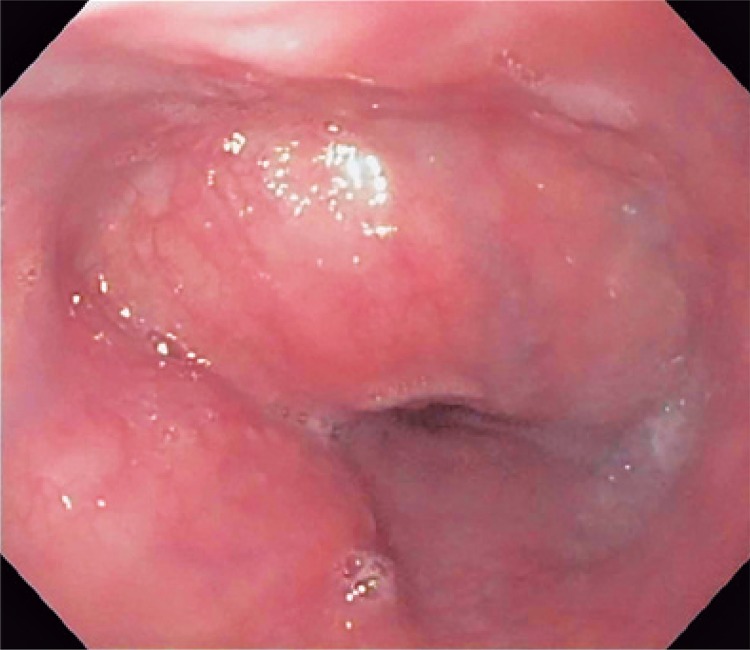
Esophagogastroduedonoscopy. Extrinsic compression of the middle esophagus

A 45-year-old male patient, hospitalized and under investigation of mediastinal mass, presenting with sudoresis, malaise, and dysphagia. Computed tomography showed eccentric parietal thickening of the middle third of esophagus, with mass effect and upstream ectasia, with no expressive increase of metabolic activity. Additionally, there are cervical, axillary, pulmonary hilar, portocaval space, and unspecific iliac lymph nodes (Figures 1 and 2).

**Figure 3:**
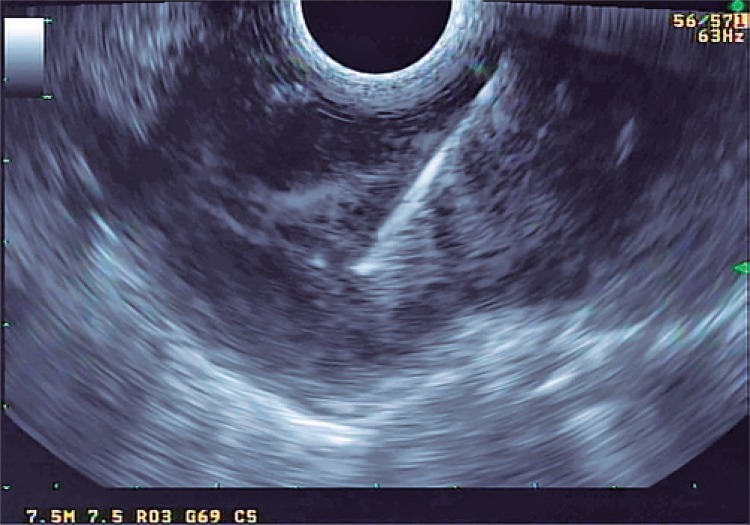
Ultrasound-guided aspiration puncture of a hypoechoic lesion of great proportions

**Figure 4:**
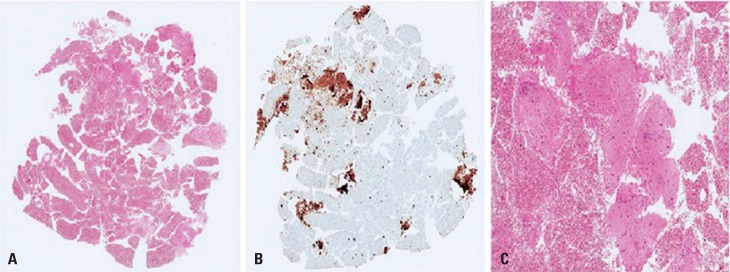
(A) Slide of the ultrasound-guided biopsy specimen (4x, hematoxylin and eosin). (B) Cells showing positive immunoexpression for desmin. (C) Magnified area showing bundles of smooth muscle cells (20x, hematoxylin and eosin)

On endoscopy, it was noted that the tumor was related to the aortic arch, left atrium, esophageal wall, and main bronchi. Transesophageal ultrasound-guided punctures were performed. Pathological and immunohistochemical studies showed fusiform cells with no mitotic activity and necrosis, with expression of smooth muscle desmin and actin, concluding the diagnosis of leiomyoma (Figures 3 and 4).

Lymphadenopathy is the most common lesion of the middle mediastinum, including lymphoma, sarcoidosis, and metastatic lung tumor.^(1)^ In this case, analyzing the age range, symptoms, and tomographic findings, the initial suspicion was of lymphoma, which was excluded only after pathological examination.

Benign tumors of the esophagus are rare, with a prevalence of up to 0.5%.^(2)^ Leiomyoma is the most common, accounting for about 60% of benign esophageal tumors.^(3)^ In general, they are small, asymptomatic lesions, which are incidentally detected in exams. In 2% of cases, they present as extraesophageal lesions, and cause compression of adjacent mediastinal structures.^(2)^ Leiomyomata are mesenchymal tumors, typically derived from the muscularis propria, but they can also originate from the muscularis mucosae. Microscopically, there are spindle-cell fascicles with no nuclear atypia. Immunohistochemistry can assist in the differential diagnosis of lymphoma, leiomyosarcoma, gastrointestinal stromal tumor, among others.^(4,5)^

Since they are usually small, asymptomatic lesions, with a benign behavior, resection is not mandatory. Clinical follow-up can be carried out by means of endoscopy. Endoscopic resection can be performed in lesions up to 2cm in size.^(6)^ Surgical treatment is reserved for symptomatic tumors, larger than 2cm or that present with image alterations or growth of more than 1cm during clinical follow-up.^(5)^

This patient was symptomatic and was referred to surgery. The surgical specimen confirmed the diagnosis.

Therefore, leiomyoma should be considered in the differential diagnosis of mediastinal tumors. Endoscopic ultrasound is an important tool for mediastinal assessment. Additionally, it is less invasive than thoracoscopy and mediastinoscopy to collect specimens.
